# A large non-inverted true diverticulum resembling a submucosal tumor of the ascending colon: a report of a rare case

**DOI:** 10.1007/s12328-025-02120-3

**Published:** 2025-04-04

**Authors:** Masahiro Maeda, Hiromichi Maeda, Kazune Fujisawa, Takayoshi Yamada, Hinako Maruoka, Ken Okamoto, Tsutomu Namikawa, Hideyuki Miyachi, Michiya Kobayashi, Satoru Seo

**Affiliations:** 1 Department of Surgery, Kochi Medical School, Kochi University, Kohasu, Oko-Cho, Nankoku, Kochi 783-8505 Japan; 2 Cancer Treatment Center, Kochi Medical School Hospital, Nankoku, Japan; 3 Department of Gastroenterology and Hepatology, Kochi Medical School, Kochi University, Nankoku, Japan; 4 Department of Pathology, Kochi Medical School, Kochi University, Nankoku, Japan; 5 Department of Clinical Nursing, Kochi Medical School, Kochi, Japan

**Keywords:** Submucosal tumor, Diverticulosis, Colon

## Abstract

A 57-year-old woman presented with a chief complaint of right lower abdominal pain. Abdominal radiography and plain abdominal computed tomography revealed a mass with extensive calcification. A colonoscopy demonstrated a 20-mm-sized mass lesion protruding into the intestinal lumen from the ileocecal valve’s lower lip. The mass was covered with normal mucosa without erosions. Endoscopic ultrasonography indicated that the tumor originated from or beneath the proper muscle layer. To treat the pain, eliminate the obstruction risk, and obtain a definitive diagnosis, the patient chose surgical resection. Laparoscopic-assisted ileocecal resection was performed under the diagnosis of a submucosal ascending colon tumor. Macroscopically, the tumor showed no gross epithelial abnormalities. However, it was filled with fecal material and had an orifice of approximately 3 mm on the proximal side of the tumor. Histopathologically, the cyst wall consisted of an entire colonic structure, and continuity was noted between the cystic lesion’s wall and the ascending colon’s wall, leading to a diagnosis of diverticular expansion due to fecal matter. No malignancies were detected. A diverticulum can collect feces and protrude into the colonic lumen, resembling colonic submucosal tumors with calcification. Although rare, this condition should be included in the differential diagnosis of colon tumors.

## Introduction

Colonic diverticulosis is a highly prevalent condition that is frequently detected during colonoscopy or computed tomography (CT), with 75–80% of cases remaining asymptomatic [1]. Diverticula often occur at sites where blood vessels penetrate the colonic walls [2]. Most cases involve multiple diverticula and pseudodiverticuloses, whereas solitary or true diverticula of the colon are uncommon. Inflammation of diverticula, known as diverticulitis, may require antibiotic treatment or surgical intervention. Otherwise, specific treatment for diverticulosis is not required. Herein, we report a case of a diverticulum filled with fecal matter protruding into the intestinal lumen and resembling a submucosal tumor (SMT), which was resected after being diagnosed as an SMT.

## Case report

**Fig. 1:**
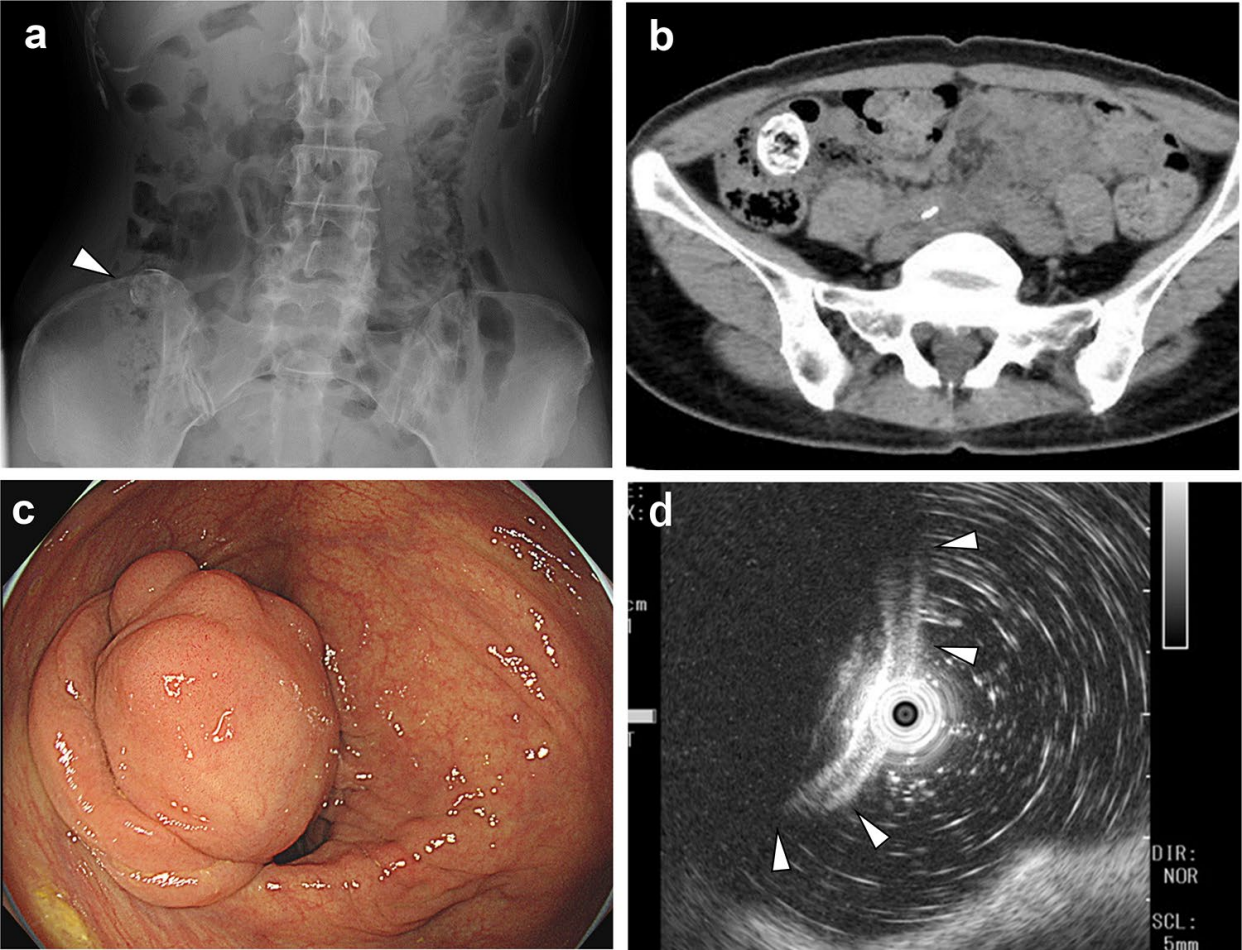
Preoperative imaging examination **a** Abdominal radiograph showing a lesion with calcifications in the right lower abdomen (white arrowhead). **b** Computed tomography image showing extensive tumor calcification. **c** A colonoscopy showed a 20-mm tumor on the ileocecal valve. The surface was covered by normal mucosa, and obstruction due to the tumor seemed likely. **d** EUS showed that the mucosa, submucosa, and muscularis layer were intact, and it was suspected that the tumor had originated from the third layer or a deeper location, but the sufficient observation was difficult due to calcification and other factors (the white arrowheads indicate the lesion covered by the normal colonic wall)

A 57-year-old woman presented with a chief complaint of right lower abdominal pain. She had a medical history of primary hyperaldosteronism, hip osteoarthritis, dyslipidemia, previous surgeries for right breast cancer, and lung adenocarcinoma. Abdominal radiography revealed a calcified tumor in the right lower abdomen (Fig. 1a). Laboratory investigations showed no significant findings, including a white blood cell count of 4.7 × 10^3^/µL and a carcinoembryonic antigen level of 1.4 ng/mL. CT revealed a 20-mm mass lesion in the ascending colon with extensive calcifications (Fig. 1b). Colonoscopy revealed a lesion protruding into the intestinal lumen from the lower lip of the ileocecal valve, which was covered by normal mucosa (Fig. 1c). Endoscopic ultrasound examination revealed normal first to third layers (Fig. 1d); we considered that the tumor originated from beneath the third layer (proper muscle layer) although the tumor was poorly observed.

Considering the right lower abdominal pain and risk of obstruction, the patient was referred to our department. Considering the diagnosis of an SMT with potential differential diagnoses of a gastrointestinal stromal tumor (GIST) or liposarcoma, the patient underwent laparoscopic-assisted ileocolic resection. Inspection of the ascending colon revealed no changes in the serosal surface of its wall. The operation time was 123 min, and the blood loss was minimal. The postoperative course was uneventful, and she was discharged on the ninth postoperative day.

**Fig. 2:**
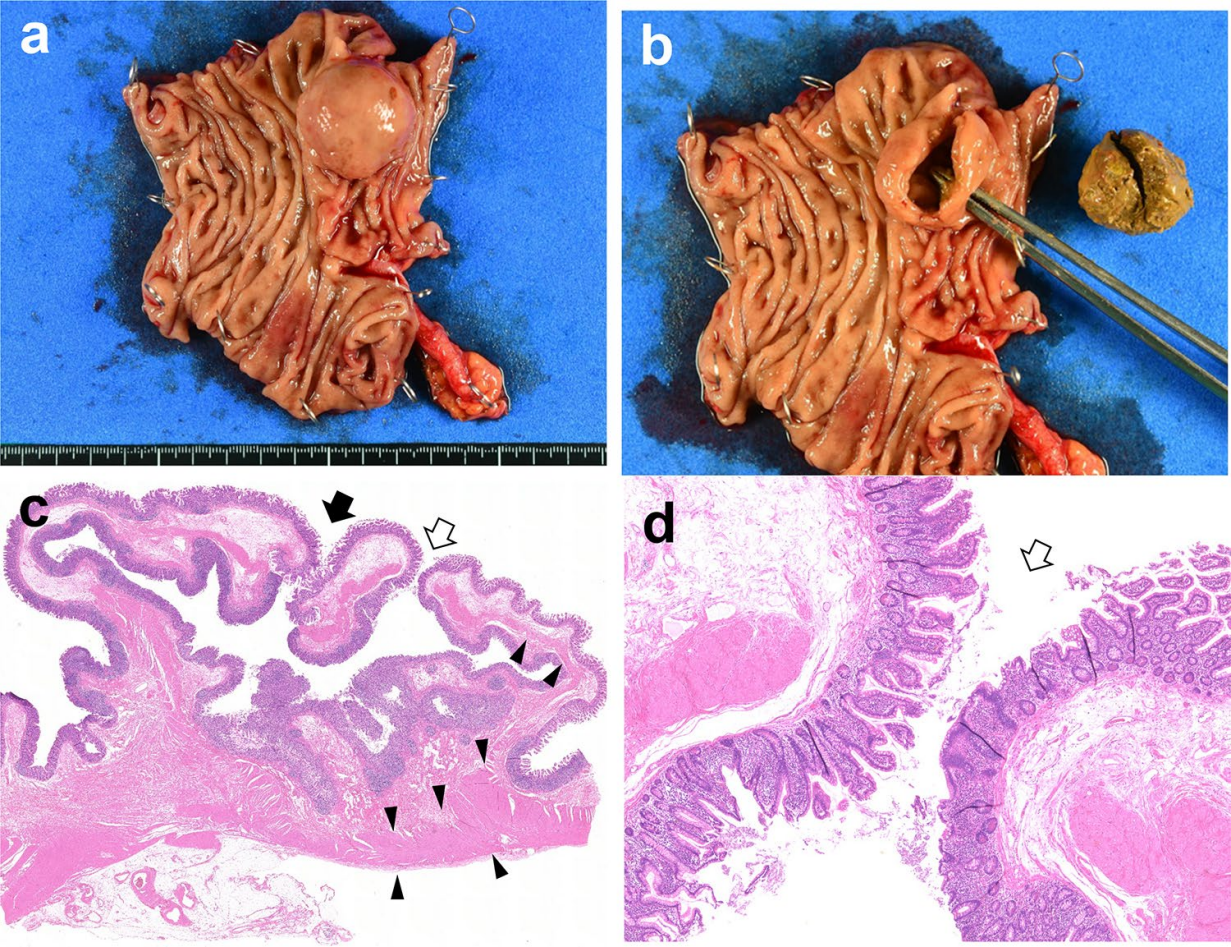
Resected tumor findings **a** The tumor was identified on the ileocecal valve **b** and was filled with fecal material. An approximately 3-mm orifice was present on the proximal side of the tumor. **c** The wall of the lesion consisted of the entire colonic wall. The proper muscle layer, which was thick at the bottom of the diverticulum and thin near the orifice, indicated the lesion to be a true diverticulum (black arrowhead indicates the muscle layers and the white arrow indicates the orifice of the mass lesion) (the black arrow indicates the site where the mass was cut to observe the internal portion after surgical resection). **d** A higher magnification of the orifice revealed the continuation of the epithelium (the white arrow indicates the orifice of the lesion)

In the excised specimen, a 20-mm tumor was identified on the ileocecal valve, with no gross abnormalities observed in the mucosal epithelium (Fig. 2a). The tumor was filled with fecal matter. An orifice of approximately 3 mm in diameter, which could not be identified preoperatively, was identified on the oral side of the tumor (Fig. 2b). Histopathological examination revealed that the cystic lesion’s wall exhibited colonic mucosa on both sides of the tumor wall. The lesion wall comprised the entire colonic wall, including the mucosal muscularis, submucosal layer, and a thin layer of muscularis propria (Fig. 2c). No heterotopic gastric mucosa or pancreatic tissue was observed. Continuity was noted between the cystic lesion’s wall and the ascending colon’s wall at the tumor orifice (Fig. 2d), leading to a diagnosis of true diverticular expansion due to fecal material accumulation. No mucosal erosions were observed; however, lymphocytic infiltration was frequently observed in the mucosal intrinsic layer to the submucosal layer, and numerous lymph follicles were formed.

## Discussion

In this case, the patient underwent surgical resection of the lesion after being diagnosed as an SMT of the ascending colon. SMTs are lesions that originate below the submucosal layer, often protruding into the gastrointestinal lumen and covered with non-neoplastic mucosa. These tumors include lipomas, carcinoids, lymphangiomas, GISTs, and malignant lymphomas. SMTs with calcification, as observed in the present case, include GISTs, liposarcomas, and rectal carcinoid tumors [3, 4]. In large GISTs, localized calcification is common and often appears circumscribed, mottled, or patchy [5]. Extensive calcification throughout most parts of the tumor has seldom been reported [6–8]. In this case, although rare, a GIST with calcifications was suspected based on similarities with the imaging features of previously reported cases.

An inverted colonic diverticulum (ICD) is a diverticular lesion protruding into the intestinal lumen. It is observed in approximately 1.7% of patients on endoscopic examinations and has a polyp-like appearance [9]. Reverting the lesion to its normal position using forceps or insufflation is diagnostic in such cases [10, 11]. Additionally, the concentric circle pattern of colonic mucosa, which becomes more evident with narrow-band imaging [12], aids the diagnosis of diverticulosis [9]. Caution is required because a biopsy or polypectomy for ICD may result in perforation. In this case, while the diverticulum protruding into the lumen resembled an inverted diverticulum, it was not an inversion but an expansion of the diverticulum toward the lumen, indicating a different pathology. In addition, colonic diverticula are often pseudodiverticula; however, in our case, the patient had a diverticulum with all layers of the colonic wall.

Inada et al. presented a rare case of a pedunculated, feces-filled, non-inverted pseudodiverticulum in the ileum [13]. Based on the lack of ectopic tissue, a muscular layer, and feces inside the diverticulum, the authors concluded that the lesion was different from an inverted diverticulum. According to the authors, such lesions rarely occur in the colon, presumably because the orifice of the colonic diverticulum is usually large enough for feces to return to the colon, and the extensibility of the colon is lower than that of the ileum. Therefore, colonic diverticula filled with feces merely enlarge and do not extend toward the lumen. We partially disagree with this hypothesis because the orifice of the diverticulum was large in our patient. Instead, we claim that the enlargement of the diverticulum is due to a balance between the extensibility and components of the colonic wall, the size of the orifice, and the viscosity of the feces. Although neither hypothesis can be further proven, we believe that our case is extremely rare. A PubMed search with the keywords “diverticulosis,” “submucosal tumor,” and “colon” did not show any similar cases. Therefore, we consider this the first reported case of a large, non-inverted true diverticulum resembling an SMT.

Intestinal duplication is a possible differential diagnosis of the present case. It has been described clinically and pathologically by Ladd and has three characteristics: (1) well-formed smooth muscle layers, (2) an epithelial lining consisting of some portion of the alimentary tract, and (3) contiguity with a portion of the alimentary tract [14]. Although the present case has these pathological features, we diagnosed a true diverticulum rather than intestinal duplication based on the following points. First, most cases of intestinal duplications are observed before the age of 2 years, and this condition is rarely observed in adults [15, 16]. Ectopic gastrointestinal epithelium is often present in intestinal duplication; however, in the present case, there was no ectopic pancreas or gastric mucosa [16]. Intestinal duplication is classified as cystic or tubular according to its shape, and the cystic lesion usually does not have communication with the normal lumen [14, 16, 17]. In the present case, the lesion is cystic in shape but has communication through a large hole with the intestinal tract. Further, the muscular layer of the wall that formed the base of the diverticulum in the present case was thin compared to the other part of the colon, which we believe is supporting evidence that the case is a diverticulum. Meanwhile, Choong et al. explained that type 2 Giant Colonic Diverticulum is a true diverticulum and considered that its origin is most likely a duplication of the intestinal tract [18]. The difference between the two entities should be discussed further.

In this case, several factors contributed to the difficulty in making a definitive diagnosis. First, this condition is rare, and we encountered such a case for the first time, making it practically impossible to recall this condition. Second, colonic diverticula typically present as multiple lesions; however, in this case, the preoperative CT images and the excised specimen revealed only a single lesion. Third, the orifice of the diverticulum was located on the proximal side of the colon, making it challenging to locate the orifice during endoscopic examination.

In the present case, her abdominal pain triggered the identification and treatment of the lesion. Pathologically, lymphocytic infiltration in the mucosal intrinsic layer and the submucosal layer was demonstrated, which may have been related to abdominal pain. Meanwhile, the protruded true diverticulum might have been tractioned toward the lumen. The pain might have been caused by such traction and the obstruction of outflow at the end of the ileum. Although surgery is often chosen for symptomatic tumors, if a colonic true diverticulum is accurately diagnosed and there are no symptoms, observation may be an option. In this regard, this case is worth reporting and true diverticulum should be included in the differential diagnosis of submucosal tumors with calcification.

We present a rare case of a colonic diverticulum filled with fecal matter that was difficult to distinguish preoperatively from an SMT. As the treatment differs, this entity should be included in the differential diagnosis of SMTs to ensure appropriate patient management.
